# Neuropsychiatric presentations of hypocortisolaemia - a literature review

**DOI:** 10.1192/j.eurpsy.2023.643

**Published:** 2023-07-19

**Authors:** K. C. Ravulapalli, N. Sud

**Affiliations:** Forensic Pyschiatry, St Nicholas Hospital, Newcastle, United Kingdom

## Abstract

**Introduction:**

Patient populations in psychiatry can have low cortisol for many reasons such as adrenalitis, secondary adrenal insufficiency and poor compliance with glucocorticoid replacement therapies. A literature review in 2005 highlighted the prevalence of neuropsychiatric features in those with low cortisol. Unfortunately, this has received little to no attention in the wider literature and hypocortisolaemia is often overlooked as a cause of neuropsychiatric presentations in clinical practice.

**Objectives:**

Review the literature to understand what psychiatric features hypocortisolaemic patients present with and any themes between cases.

**Methods:**

A literature review on neuropsychiatric presentations of hypocortisolaemia was performed using PUBMED and Google Scholar. English language studies from 2005 to October 2022 were included and searched for using the following term: (Hypocortisolaemia* OR “Low-cortisol” OR Addisons OR Adrenal-crisis OR “Adrenal insufficiency”) AND (Psychiatric OR Hallucination OR Neuropsychiatric OR “Neuropsychiatric symptoms” OR Pyschosis*). Citations in relevant papers were also reviewed.

**Results:**

7 case reports were identified, 5 male (71%) and 2 female (29%) with an average age of 42 (28-63). The cause was identified as Addisons’ disease in 4 patients (57%) and secondary adrenal insufficiency in 3 patients (43%). Hallucination or delusion was the most prevalent symptom with 86% of patients initially presenting with it, followed by depression (43%) and speech abnormality (14%). In all cases, basic blood sets (Full blood count, urea & electrolytes and liver function tests) were done in an initial assessment. 6 patients presented with hyponatraemia, and 4 of these patients had hyponatraemia as their only abnormality within their U&E profile. In one patient this delayed their diagnosis by several years. One patient developed psychosis again when being treated with glucocorticoid therapy. In 4 patients, adrenal pathology was not suspected and cortisol was not tested until initial differentials were investigated and ruled out.

**Conclusions:**

Further case reports highlight psychosis being a key feature of hypocortisolaemia that presents initially with neuropsychiatric symptoms. Cortisol levels should be considered in initial investigations of psychosis if hyponatraemia is discovered even in the absence of hyperkalaemia to help aid an earlier diagnosis. A rebound psychosis may be seen once starting glucocorticoid therapy. Additionally, there should be consideration of neuropsychiatric monitoring in stable psychiatric patients undergoing cortisol treatment.

**Disclosure of Interest:**

None Declared

